# The Analysis of the Innovation Consciousness of College Student Entrepreneurs Under the Teaching Concept of Chinese Excellent Traditional Culture

**DOI:** 10.3389/fpsyg.2021.717336

**Published:** 2021-11-15

**Authors:** Bin Geng, Tianyun Huang, Xinsheng Jiang, Nana Lin, Guangyuan Gao, Lisha Fan

**Affiliations:** ^1^Department of Wushu and Chinese Traditional Sportology, Physical Education College of Zhengzhou University, Zhengzhou, China; ^2^ICO NIDA, National Institute of Development Administration, Bangkok, Thailand; ^3^School of Educational Science, Hunan Normal University, Changsha, China; ^4^Arts Department Heilongjiang International University, Harbin, China; ^5^Department of Drama, Art College, Cheongju University, Cheongju, South Korea

**Keywords:** traditional culture, college student entrepreneur, questionnaire, innovation and entrepreneurship, Cobb-Douglas

## Abstract

The purpose of this study was to analyze the current situation of the entrepreneurial consciousness of college student entrepreneurs and to explore the role of innovative and entrepreneurial talents in social and economic development. Based on the teaching concept of Chinese excellent traditional culture, first, the relevant theories of innovation and entrepreneurship, as well as the characteristics of entrepreneurial talents in colleges and entrepreneurs, are analyzed and elaborated; moreover, the definition of college student entrepreneur is explained; then, from the perspective of entrepreneurial teaching management, entrepreneurial education, and place support, the questionnaire method is selected to show the understanding of the entrepreneurship of college students; finally, based on the Cobb-Douglas function, the model before and after the introduction of innovative and entrepreneurial talents is tested and analyzed. Investigation and analysis suggest that most college students have entrepreneurial intention, and 61.5% of them choose to start their own business after having working experience; the relative freedom of time and space is the main factor to attract college students to start their own businesses, accounting for 42.3%; 69.3% of college students think that capital is a restricting factor for entrepreneurship, while 76.2% think that lack of experience is a major restricting factor for entrepreneurship; college students have a certain demand for entrepreneurship training and guidance from the school, especially in the setting of entrepreneurship incubation park and resource pool; the characteristics of entrepreneurship, professional skills, and interpersonal resources are more crucial for college students; most college students have a positive cognition of the excellent traditional Chinese teaching concepts; the analysis based on the Cobb-Douglas function reveals that the introduction of innovative and entrepreneurial talents can promote economic development. This exploration has a positive effect on the cultivation of awareness of college students of entrepreneurship and innovation, as well as the relationship discussion between the introduction of innovative and entrepreneurial talents and social economy.

## Introduction

The growth and development of college students are closely related to the development of society and even a country. In recent years, the requirements for innovation have been constantly improved. No matter which country or industry, entrepreneurship is an aspect that cannot be ignored in promoting social development, which is of great significance to the economic development of the country and society (Teece, 2017; Haruyama and Hashimoto, 2020). In the last century, economic growth and innovation technology development are inseparable, and the innovation at that stage even led to the global economy. At present, the form of innovation is more abundant, and its role in economic development becomes more and more obvious. Encouraging innovation has become a regular and long-term policy (Yang, 2019; Ruii et al., 2021). Influenced by the current social environment and the impetuous market environment, the perception of traditional culture of college students is not so clear. Therefore, it is necessary to constantly improve the recognition of traditional culture by this special group (Shi et al., 2019). The Chinese excellent traditional culture contains rich contents, and attention should be paid to the education of contemporary youth culture perception and identity. The rapid development of artificial intelligence and mobile Internet has brought many new enterprises; however, many new ventures have disappeared, among which the reasons are relatively complex (Danilin, 2020; Lu et al., 2020; Pena et al., 2021). In view of innovation and entrepreneurship, Wu et al. (2019) selected MBA majors of the Tianjin University as the research object and analyzed and discussed the entrepreneurial intention of students based on their self-efficacy, which provided some reference for promoting entrepreneurship education and improving entrepreneurial practice activities. Buenstorf et al. (2017) selected college dropouts as the research object and analyzed the relationship between college dropouts, graduation, innovation and entrepreneurship activities, and entrepreneurial performance. The majority of those who have dropped out of school decided to start a business. However, even if people become entrepreneurs, they still need to adapt to the fierce market competition environment. Stel et al. (2017) studied and analyzed the relationship between entrepreneurial motivation and entrepreneur income. The results suggest that no matter what the type of necessary motivation is, the income of corresponding entrepreneurs is significantly lower than that of opportunistic entrepreneurs, and this difference runs through the whole process of entrepreneur tenure and has permanent characteristics. Ahsan et al. (2018) analyzed the challenges faced by student entrepreneurs in the process of entrepreneurship. Based on the social cognitive theory of self-regulation, a two-stage model was constructed to analyze the effect of the emotional state of student entrepreneurs on entrepreneurial identity and its process. It was found that the dual role of effective guidance combined with the model founders is conducive to the transition from students to entrepreneurs (Ahsan et al., 2018). Moreover, there is a lot of research work on the entrepreneurship of college students. According to previous studies, the innovation and entrepreneurship activities and intentions of the college students are affected by multiple factors, and the transition from students to entrepreneurs is also affected by multiple factors. Throughout the existing research, it was found that the research work of integrating the teaching concept of traditional culture into it is not much, and the research work focusing on individual college students is also relatively scarce.

Based on this, this exploration aims to analyze the current situation of the entrepreneurial consciousness of college students and to discuss the impact of the introduction of innovative and entrepreneurial talents on the social economy. With college students as the research object, through the method of questionnaire combined with model test, the factors influencing the innovation and entrepreneurship of college students are summarized, and the role of college students as innovative and entrepreneurial talents (i.e., college student entrepreneur) in social and economic development is simply analyzed. The research innovation is that the role of the teaching concept of Chinese excellent traditional culture is considered in the analysis of the current situation of the entrepreneurial consciousness of college students, and the preliminary analysis and discussion on the relationship between the introduction of innovative and entrepreneurial talents and the social and economic development is made.

## Innovation and Entrepreneurship, Talent Training, and Entrepreneur Characteristics

### Teaching Concept of Chinese Excellent Traditional Culture

After years of baptism and inheritance, the Chinese excellent traditional culture covers all levels of content, such as literature and technology in the traditional sense, and has the complementary unity between the spiritual connotation and the realization carrier. In terms of spiritual connotation, it reflects the relationship between man and nature, country, society, and themselves. The so-called survival concept, family and country feelings, social care, and personality cultivation are the core embodiment and representation of its spiritual connotation; from the carrier of realization, the physical and cultural image, such as language, words, brilliant art, and science and technology, is the core embodiment of its carrier of realization. In terms of relevance, the former is the embodiment of the inner temperament of the latter, while the latter is the external manifestation of the former. In higher education, cultural identity plays an important role in the formation of the teaching mode. The realization of cultural identity depends on rational cognition, emotional identity, belief guidance, and practical reinforcement of the related objects. For the special group of college students, cultural identity is also reflected in the concept and behavior of innovation and entrepreneurship (Chan, 2017; Ward et al., 2021). In many speeches, General Secretary Xi also stressed the importance of Chinese traditional culture and its significance in establishing world outlook, outlook on life, and values. Under the background of the new era of mass entrepreneurship, the innovation and entrepreneurship ideas contained in the Chinese excellent traditional culture undoubtedly play a positive role in promoting the inheritance of culture and promoting innovation and entrepreneurship education. Therefore, relevant analysis is made based on the teaching concept of Chinese excellent traditional culture.

### Related Theories of Entrepreneurship and Innovation

The rapid development of the economy and the aggravation of market competition put forward higher requirements for talent training and innovation. The definition of innovation is very different. No matter what the new way of understanding is, the premise of “new” remains unchanged (Wu and Song, 2019). Based on innovation, social economy and creativity also develop and progress in this process (Januzzi and Ibrahim, 2020; Wang and Hou, 2020; Aldianto et al., 2021). The concept of innovation is first put forward in the book *Economic Development Theory*. At this time, entrepreneurs are considered to be the core of innovation, and new technologies or methods are considered to be the embodiment of core content. Later, on this basis, institutional innovation is developed; then, the understanding of innovation is further expanded and extended, which is no longer confined to technology and system, but involved many aspects such as school and humanistic quality, from which the theory of systematic innovation comes into being. Similar to innovation, entrepreneurship has also experienced different levels and degrees of development. The so-called entrepreneur refers to the leader and coordinator in the specific production practice; for this, some people think that entrepreneurship is an indispensable prerequisite for innovation; others study the characteristics of entrepreneurial psychology. Cultural identity is also a component of social cognition, and the social environment has a great influence on entrepreneurs (Engineer et al., 2019; Abraham, 2020).

Based on the abovementioned content, it is easy to find that for the entrepreneurial groups of college students, the recognition of Chinese excellent traditional culture runs through the whole process of innovation and entrepreneurship.

### Entrepreneurial Talents and Entrepreneur Characteristics in Colleges

The entrepreneurship of college students has always been concerned. In view of this, the government has also issued relevant supporting policy documents, which are enough to show that the state attaches great importance to the entrepreneurship of college students. It is a possible choice for graduates to promote employment through entrepreneurship. Among them, the talent training mode of colleges naturally occupies a key proportion, which is also a basic problem in higher education. This exploration holds that the talent training mode is that colleges, as the main body of undertaking the educational task, can help college students to construct the structure of knowledge and quality, so as to achieve the purpose of cultivating innovative and entrepreneurial talents.

Based on the abovementioned entrepreneurial theory, the so-called entrepreneurs are those who make efficient use of social resources based on the construction of new enterprises. Through the reorganization and integration of relevant production factors, entrepreneurs can achieve the creation of new value. It can be said that entrepreneurs are the participants of social-related economic activities (Rogoza et al., 2018). For the entrepreneurial talents of college students, it is believed that they are based on innovation ability, have entrepreneurship, have certain entrepreneurial qualities (EQ), are good at seizing opportunities, can take corresponding risks, and realize their own value-added through the establishment of enterprises. These people are called college student entrepreneurs.

**FIGURE 1:**
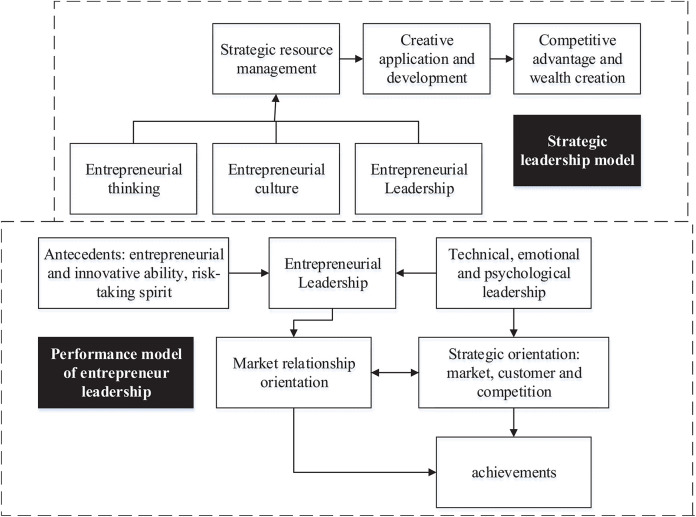
Strategic model and performance model of entrepreneur leadership.

Entrepreneurship is influenced by multiple factors, such as social culture, economic system, and individual factors (Tanner et al., 2019). This exploration focuses on the influence of Chinese excellent traditional culture on entrepreneurship. To become a new entrepreneur, entrepreneurial leadership is one of the necessary elements (Qian et al., 2018; Chen, 2019; Chang and Chen, 2020), among which the view of characteristics and ability, the view of the behavior, and the view of the process are the three main manifestations. Figure 1 presents the strategic model and performance model of entrepreneurial leadership. From the perspective of entrepreneurial leadership, the strategic model of entrepreneurs mainly includes entrepreneurial thinking, entrepreneurial culture, and entrepreneurial leadership. Innovative development and application are carried out based on that, which reflects a certain competitive advantage, so as to achieve the creation of wealth. The performance model is closely related to the strategic model. Innovation ability, risk-taking spirit, technology, psychological emotion, and moral aspects are the key influencing factors of entrepreneurial leadership. Then, customer orientation, competition orientation, and collaboration will affect market relationship orientation. Finally, they will exert a certain impact on enterprise achievements under the role of entrepreneurial leadership and strategic orientation.

**FIGURE 2:**
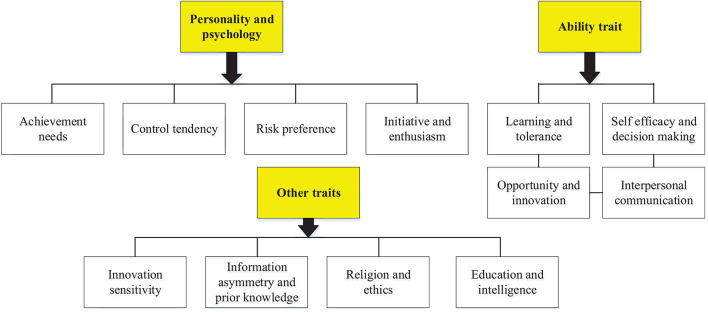
Characteristics of entrepreneurs.

Figure 2 shows the specific characteristics of entrepreneurs. It reveals that achievement demand, control tendency, risk preference, initiative, and passion are the main personality and psychological characteristics of entrepreneurs, while learning ability, tolerance, opportunity innovation, and interpersonal communication are the main characteristics of entrepreneurs. Besides, entrepreneurial agility, information asymmetry, prior knowledge, education, and intelligence are also the embodiment elements of entrepreneurial characteristics.

To analyze the cultivation of the entrepreneurial consciousness of college students from the perspective of the Chinese excellent traditional culture, a city in Shanxi Province is taken as the research object. By means of the questionnaire, the current situation of talent work and the entrepreneurial talent demand in the city are analyzed.

## Questionnaire Design and Model Selection

### Questionnaire Design

For the support of colleges for entrepreneurship teaching management and entrepreneurship education of college students, and places under the teaching concept of Chinese excellent traditional culture, the way of questionnaire survey is mainly selected to carry out the corresponding analysis (Seitzinger et al., 2019). The main purpose of this questionnaire is to understand the overall situation of entrepreneurship of college students and to analyze the role of the teaching concept of Chinese excellent traditional culture in college students. The distribution of the questionnaire is mainly completed through the “questionnaire star” platform, mainly aimed at junior and senior undergraduates. The selection of the research object involves different genders, family backgrounds, majors, regions, schools, and so on, which has a wide coverage and plays a positive role in the research development. The research results are universal to a certain extent. To ensure the effective recovery of the questionnaire, the teachers of the entrepreneurship center of college students are contacted in advance before the questionnaire is issued. A total of 200 questionnaires are distributed, and 180 questionnaires are finally collected, with an effective rate of 90%. The reliability and validity of the questionnaire collected are analyzed by SPSS 25.0 (IBM, United States) (Nunfam et al., 2021). Cronbach’s alpha is used to analyze the reliability, and the Kaiser-Meyer-Olkin (KMO) test and Bartlett’s test are adopted to analyze the validity. It is found that the designed questionnaire had good reliability and validity. The purpose of this questionnaire is to analyze the overall situation of the entrepreneurship of college students. Table 1 reveals several key issues involved in the questionnaire design. Specifically, the questionnaire mainly includes five dimensions, namely, entrepreneurial inducing (EI) factors, entrepreneurial limiting (EL) factors, entrepreneurial supporting (ES) factors, traditional culture teaching ideas (TCTI), and EQ.

**TABLE 1:** Key question setting of the questionnaire.

**Item distribution**	**Content and specific options**		
Q6	C1: Do you choose to start a business when you are in school or after graduation?	C11: Entrepreneurship is already in progress	
		C12: Choose to start a business after graduation	
		C13: Choose to start a business after having working experience	
		C14: Do not choose to start a business	C14.1: Work is the premise
			C14.2: No work
Q9	C2: What are the main reasons for your entrepreneurial ideas?	C21: Supported by relevant policies	
		C22: Suggestions from people around	
		C23: Solve the difficult problem of employment	
		C24: Have good ideas and want to put them into practice	
		C25: Get more income	
		C26: Work with more freedom, less constrained, more free time	
Q10	C3: What do you think are the main reasons for limiting the entrepreneurship of college students?	C31: No start-up funds	
		C32: Lack of experience	
		C33: Lack of knowledge and skills for entrepreneurship	
		C34: A few good projects and lack of human resources	
		C35: Relevant entrepreneurship policy support is not in place, lack of public service resources	
Q12	C4: What kind of help do you want schools to provide fo entrepreneurship?	C41: Strengthen the training of entrepreneurship education	
		C42: Build resource bank and set up a business incubator	
		C43: Strengthen the publicity of relevant policies	
		C44: Strengthen school-enterprise cooperation and provide professional advice and guidance	
		C45: Support and encourage entrepreneurship	
Q14	C5: What do you think of the excellent traditional culture teaching mode?	C51: Traditional culture teaching mode has a positive guiding role for entrepreneurship	
		C52: Under the background of the development of the Internet and artificial intelligence, it has little effect	
		C53: Unclear	
Q15	C6: What do you think of the influence of traditional culture teaching concepts on entrepreneurship?	C61: It has a positive impact C62: It has a negative impact C63: No opinion	
Q16	C7: What do you think of the characteristics that college students should have as entrepreneurs?	C71: Have an entrepreneurial spirit	
		C72: Have flexible thinking and have their own understanding of a certain field	
		C73: Have good interpersonal relationship and resources	
		C74: Have support from family or school	

### Empirical Analysis Based on Innovative and Entrepreneurial Talents

College student entrepreneurs are the core force of innovative and entrepreneurial talents. Based on the questionnaire, to analyze the relationship between innovative and entrepreneurial talents of college students and the social and economic development and to further analyze the current situation and development of the entrepreneurial consciousness of college students, the Solow model of Cobb-Douglas is introduced for the analysis (Akaev and Sadovnichii, 2019; Germain, 2019; Zhang et al., 2020). The corresponding equation is as follows:

(1) $$Y=E\,\left(t\right)\,F\,A\,I_{t}^{\alpha }\,L\,I_{t}^{\beta }\,e^{R\,E}$$

Equation 1 represents gross domestic product (GDP), capital input, labor input, efficiency constant, parameters to be estimated, and random error.

Equation 1 can further be evolved into the following:

(2) $$L\,n\,Y=L\,n\,E\,\left(t\right)+\alpha \,L\,n\,F\,A\,I_{t}+\beta \,L\,n\,L\,I_{t}+R\,E$$

By further subdividing into ordinary input and talent input, Eq. 1 can be changed into the following:

(3) $$Y=E\,\left(t\right)\,F\,A\,I_{t}^{\alpha }\,L\,I_{t}^{*\beta }\,I\,E\,T_{t}^{\gamma }\,e^{R\,E}$$

Equation 3 represents the input of ordinary labor and the input of talents. Logarithms are taken on both sides of the equation, and it can be transformed into the following:

(4) $$L\,n\,Y=L\,n\,E\,\left(t\right)+\alpha \,L\,n\,F\,A\,I_{t}+\beta \,L\,n\,L\,I_{{}^{t}}^{*}+\gamma \,L\,n\,I\,E\,T_{t}+R\,E$$

Considering the definition of the term “innovation” and entrepreneurship talents and the complexity of the data collection and acquisition, the professional and technical talents are mainly analyzed, that is, those with a certain professional and technical level and strong innovation ability, who are distributed in all walks of life. On this basis, through the method of regression analysis, a general analysis of the relationship between the entrepreneurship of college students and the social economy is made. Table 2 reveals the independent variables, dependent variables, and their output indexes involved in the regression analysis.

**TABLE 2:** Composition of regression analysis indexes.

Independent variable	College student entrepreneurs, fixed assets input, and labor input
Dependent variable	Social and economic development
Specific output indexes	The coefficient of determination, multiple correlation coefficient, standardized coefficient, *t*-test value, and variance test value before and after adjustment

## Results of Questionnaire and Model Test

### Analysis of Questionnaire Results

**FIGURE 3:**
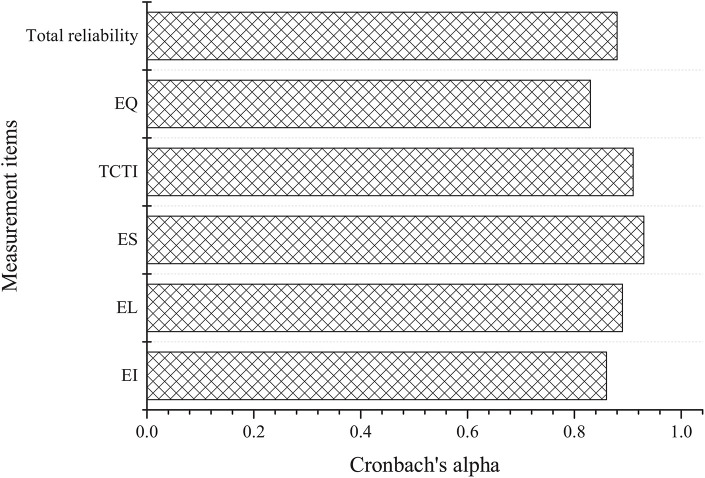
Results of the reliability analysis of the questionnaire.

Figure 3 and Table 3 display the results of the reliability and validity analysis of the questionnaire, respectively.

**TABLE 3:** Results of the validity analysis of the questionnaire.

**Key dimensions of the questionnaire**	**KMO**	**Bartlett’s test**	**Significance**
EI	0.93	4632.94	0.000
EL	0.83	1849.34	0.000
ES	0.88	1748.56	0.000
TCTI	0.85	1872.75	0.000
EQ	0.86	1948.24	0.000

The data analysis of Figure 3 and Table 3 shows that the questionnaire has good reliability. The Cronbach’s alpha values of each key dimension are above 0.8, and the total reliability of the questionnaire is 0.88; regarding the validity, the KMO test values of each key dimension of the questionnaire are above 0.8, and each dimension shows good significance, which shows that the validity is good. Overall, the questionnaire has good reliability and validity.

**FIGURE 4:**
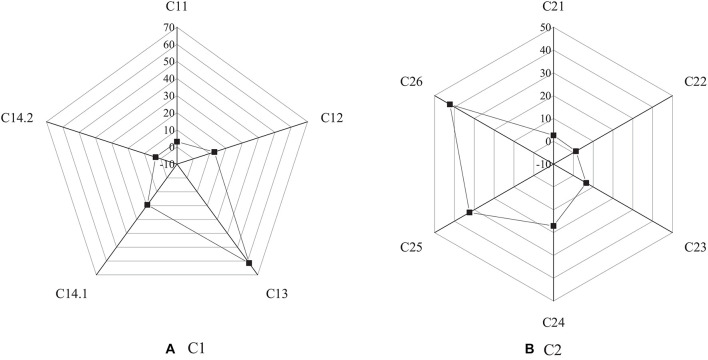
Survey results based on options **(A)** C1 and **(B)** C2.

**FIGURE 5:**
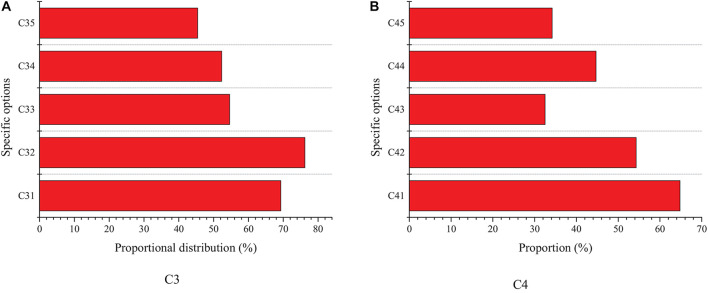
Survey results based on **(A)** C3 and **(B)** C4.

**FIGURE 6:**
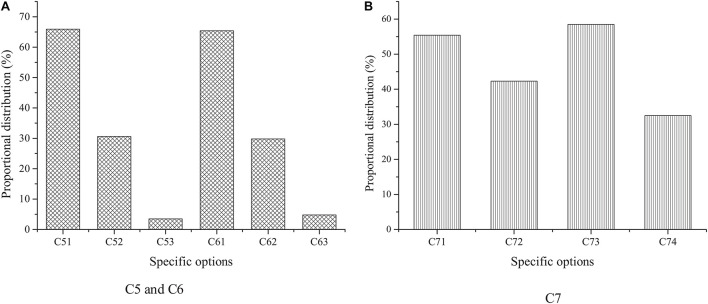
Survey results based on **(A)** C5 and C6 and **(B)** C7.

Figures 4–6 show the survey results of several key items in the questionnaire. Figure 3 shows the survey results based on C1 and C2 options.

The survey results of C1 show that among college students, the proportion of those who have started a business is the least, accounting for 3.1%; most of the college students have great intention to start a business. Among them, 61.5% of them are interested in starting a business after having work experience, while 12.9% of them are interested in starting a business after finishing their studies. It suggests that the intention of college students for entrepreneurship is larger. The survey results of C2 show that 42.3% of the people choose the option “work with more freedom,” and 32.4% choose the option “get more income.” Among many factors, 17.1% of them choose to put their creative ideas into practice, and 6.5% of them think it is difficult to find jobs. The data analysis reveals that most of the college students have the intention to start a business. It indicates that entrepreneurship has a certain attraction for college students, among which most of them hope to start a business after accumulating certain work experience. Most people think that entrepreneurship can have more free time and space, and the current severe employment situation has become a crucial reason for college students to choose entrepreneurship.

Figure 5 shows the survey results based on C3 and C4 options.

The survey results of C3 suggest that the proportion of students who think that the funds are insufficient and the experience is insufficient is the largest, among which the proportion of the former is 69.3%, the latter is as high as 76.2%; the proportion of students who think that the professional knowledge or skills are insufficient is 54.6%, and the proportion of students who choose the reason of lack of resources is 52.3%. The survey results of C4 reveal that college students hope that the help provided by the school is to strengthen training and to set up a business incubator and its entrepreneurial resource pool. In short, it is found that capital and experience are the main factors restricting or promoting the entrepreneurship of college students, and most people regard resources as the main factors that affect whether they choose to start a business. Besides, the expectation of support of college students from the school level is particularly urgent, which shows that the demand for entrepreneurial professional knowledge and skills is very high.

Figure 6 shows the survey results based on C5–C7 options.

The results of C5 and C6 indicate that most students have a relatively positive attitude toward the traditional culture teaching mode and think it is beneficial to the cultivation of innovation and entrepreneurship awareness; moreover, most college students think that the traditional culture teaching concept has a positive impact on entrepreneurship. The survey results of C7 show that the proportion of students who choose the entrepreneurial spirit, professional skills, and human resources is more. On the whole, the traditional culture teaching has a certain influence among college students, which also reflects the recognition of college students for the excellent traditional Chinese culture, and their expectations for it to play a positive role in their own entrepreneurship.

After the results of the questionnaire at all levels are integrated, it is easy to find that college students have a great intention to start a business. However, due to the influence of various factors, most college students do not regard entrepreneurship as the current choice. More free working time and space are the factors that are more popular with college students and one of the key factors to attract college students to start their business. It is not difficult to find that among the key factors limiting the entrepreneurship of college students, most people have the most concerns about the entrepreneurial experience, ability, start-up capital, professional skills, and so on. With entrepreneurship education as the background and entrepreneurship intention as the research object, Huang et al. (2021) found that the support of entrepreneurship policy and entrepreneurship practice exerts a great impact on the entrepreneurship intention of college students, which can promote the entrepreneurship and innovation behavior of college students to a certain extent. This is consistent with the research results obtained from this exploration. Students expect schools to provide more support and training in entrepreneurship and professional skills. In terms of the traditional culture teaching concept, most students still maintain the attitude of recognition and think that the traditional culture teaching concept has a certain promotion effect on the cultivation of the entrepreneurial ability of students. In this regard, Bogatyreva et al. (2019) analyzed the differences among the entrepreneurial behaviors of college students and finally concluded that the core level of national culture exerts a significant impact on the relationship between entrepreneurial intention and subsequent behavior. At present, there is not much research on the relationship between the Chinese excellent traditional culture and the entrepreneurship of college students, but it provides more powerful support for the research work of this exploration. Moreover, it verifies the effectiveness of this exploration. The abovementioned results are consistent with the actual situation. College students, a special group, are in a critical period of physiological and psychological development. The increasingly severe employment situation makes entrepreneurship an effective way. It is also one of the factors influencing the entrepreneurial intention of college students. In addition, the influence of traditional culture on college students is subtle, and the role of this soft power component cannot be underestimated. The assistance of such auxiliary factors is reflected in the whole process of innovation and entrepreneurship.

### An Analysis of the Relationship Between College Student Entrepreneurs and Economic Development

**FIGURE 7:**
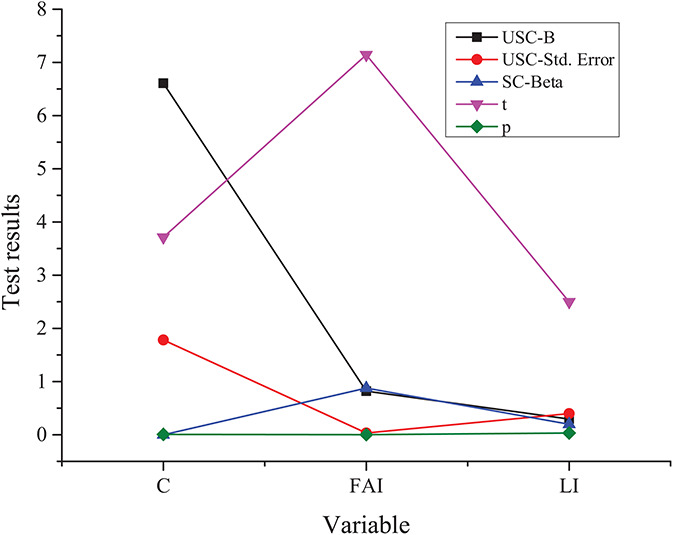
Test results based on the Solow model. C, constant term; FAI, the input of fixed assets; LI, labor input; USC, the non-standard coefficient; SC, standard coefficient.

The relationship between the innovation and entrepreneurship talents among college students and the social and economic development is analyzed based on the Solow model. Figure 7 presents the final results.

The analysis of relevant data changes in Figure 6 shows that the Solow model obtained from the corresponding data of the statistical analysis among economic development, capital input, and labor input has passed the test, and the final goodness of fit is relatively high. Specifically, it can reach 0.996. In addition, the estimation coefficient test of the corresponding constant term and independent variable also shows good fitting results. Specifically, the significance and confidence levels are at a high level, and the latter can reach 96%. In conclusion, there is a Cobb-Douglas relationship between economic development, capital input, and labor input.

**FIGURE 8:**
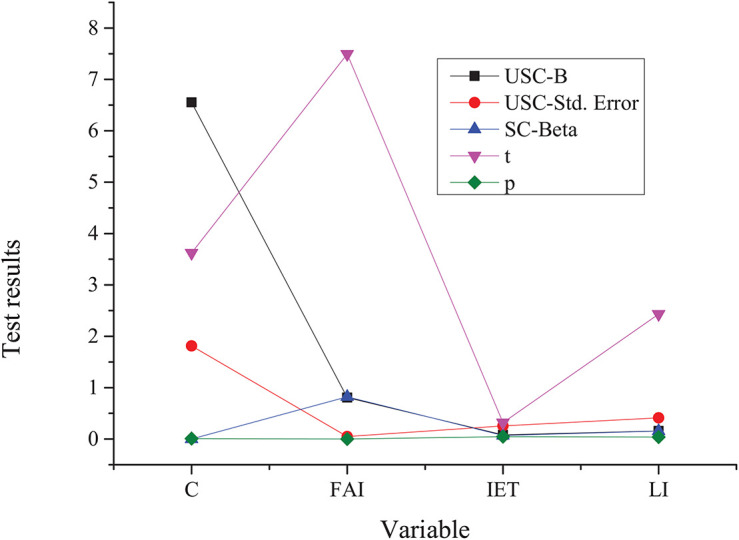
Model test results after introducing innovative and entrepreneurial talents. IET, innovative and entrepreneurial talents.

Figure 8 shows the analysis result based on the special innovative and entrepreneurial talents of college students.

The analysis of relevant data changes in Figure 7 shows that the economic development has good goodness of fit with the input of capital, labor force, and innovation and entrepreneurship talents, which can reach 0.996 in numerical terms. Based on the constant term and fixed assets input (FAI), the confidence level of the estimated coefficient is as high as 98%. It indicates that economic development, capital, innovation and entrepreneurship talents, and labor input still meet the Cobb-Douglas after the inclusion of innovation and entrepreneurship talents. The elasticity coefficient corresponding to innovation and entrepreneurship talents can reach 0.069, while the elasticity coefficient corresponding to the conventional labor force can reach 0.157. When innovation and entrepreneurship are introduced, the elasticity coefficient of fixed asset investment decreases, while investment exerts a minimal effect on economic development.

Generally, the use of the Cobb-Douglas function can effectively analyze the relationship between input factors of regional economic development from different levels. The introduction of innovative and entrepreneurial talents has a certain impact on all levels. The data analysis results show that talent introduction has a positive role in promoting economic development, even if the number of people introduced is not large. Jiang et al. (2020) found that the aggregation of high-quality innovative talents will exert a greater impact on urban innovation, and the sustainable development of the urban economy is also affected by innovative and entrepreneurial talents. Hence, it is essential to strengthen the cultivation of the entrepreneurship consciousness of college students from the school level, which has a positive effect on the economic development of the region and even the whole society. Undoubtedly, the introduction of innovative and entrepreneurial talents will greatly influence the social and economic development of a region. The gathering of high-quality innovative talents is a crucial driving factor to lead the regional industrial and economic development. More people with entrepreneurial spirit and entrepreneurial characteristics can promote economic development to a certain extent.

## Conclusion

The innovation- and entrepreneurship-related theories and entrepreneurial characteristics are analyzed based on the excellent traditional Chinese teaching concept. Then, the questionnaire combined with the model test is adopted to analyze the current situation of the entrepreneurial consciousness and related elements of the college students. Finally, the relationship between entrepreneurship and economic development is evaluated based on the Cobb-Douglas function. The results show that college students have higher entrepreneurial intention, and the proportion of people who focus on work experience is higher. The relatively free time and space is a crucial factor to attract college students to start businesses. The lack of funds and experience is the main factor to restrict college students to start businesses. College students have an urgent need for relevant support from the school and have a positive understanding of the relationship between the teaching concept of Chinese excellent traditional culture and the entrepreneurship. The introduction of innovative and entrepreneurial talents has a certain role in promoting economic development, even a small number of the entrepreneurial entrepreneurs of college students can also bring a greater impact. This provides a certain reference for the cultivation of the innovation and entrepreneurship awareness of college students.

There are still some deficiencies. Due to the limitations of the experimental conditions, based on the introduction of students and talents, the selected research samples are difficult to achieve comprehensive coverage in the analysis of the entrepreneurial consciousness of college students. Moreover, based on the Cobb-Douglas function, regarding the research topic, only the single impact of talent introduction is evaluated, and there is no more in-depth discussion. Hence, in the future research work, the coverage of the research sample will be expanded, and a more detailed discussion of the impact of different components on the innovation consciousness of the entrepreneurial entrepreneurs of college students will be conducted. For example, a more in-depth and detailed analysis and discussion on the EI factors will be made, so as to put forward more effective suggestions and countermeasures in the cultivation of the entrepreneurial entrepreneurs of college students.

## Data Availability Statement

The raw data supporting the conclusions of this article will be made available by the authors, without undue reservation.

## Ethics Statement

The studies involving human participants were reviewed and approved by Physical Education College of Zhengzhou University Ethics Committee. The patients/participants provided their written informed consent to participate in this study. Written informed consent was obtained from the individual(s) for the publication of any potentially identifiable images or data included in this article.

## Author Contributions

All authors listed have made a substantial, direct and intellectual contribution to the work, and approved it for publication.

## Conflict of Interest

The authors declare that the research was conducted in the absence of any commercial or financial relationships that could be construed as a potential conflict of interest.

## Publisher’s Note

All claims expressed in this article are solely those of the authors and do not necessarily represent those of their affiliated organizations, or those of the publisher, the editors and the reviewers. Any product that may be evaluated in this article, or claim that may be made by its manufacturer, is not guaranteed or endorsed by the publisher.
